# Using of Chêneau brace for early onset scoliosis treatment

**DOI:** 10.1186/1748-7161-8-S1-O53

**Published:** 2013-06-03

**Authors:** D Chekryzhev, A Mezentsev, D Petrenko

**Affiliations:** 1 Sytenko Institute of Spine and Joint Pathology, Orthospine LTD, Kharkiv, Ukraine

## Background

Casting, halo-traction [1,2], Milwaukee or TLSO braces [1,3] are routinely used for the conservative treatment of early onset scoliosis (EOS). Some authors use brace only in a case of congenital scoliosis absence.

## Aim

The purpose of this study is to assess outcomes of Chêneau brace use in EOS patients.

## Methods

This is a prospective study of clinical outcomes in 17 EOS patients treated with Chêneau brace. Mean age was 4.3 years old. Follow-up was 2-7 years. There were 3 males and 14 females. 3 patients had mixed spinal anomaly (1st group), 10 patients had wedge hemivertebra (2nd group) and 4 patients had infantile idiopathic scoliosis (3rd group).

## Results

Before treatment initiation, Cobb angle in the 1st group was 52.3° (43°-60°), in the 2nd group 33.2° (25°-48°), and in the 3rd group 40° (24°-75°). After 6 years follow-up on the 1st group, 1 patient had 7° correction, 1 patient had 8° progression and 1 patient had stable deformity. In the 2nd group, mean correction was 3° (-2°– 15°) and 8.75° (0°-30°) in the 3rd group.

## Conclusion

Using a Chêneau brace results in getting EOS correction in 7 (41%) cases, stabilized deformity in 8 cases (47%), and in 2 (12%) cases progression was delayed. The results are the best we get in patients with infantile idiopathic scoliosis.

## References

[B1] Fletcher ND Larson AN Richards BS Johnston CE Current treatment preferences for early onset scoliosis: a survey of POSNA members J Pediatr Orthop 31 3 326 330 21415695 10.1097/BPO.0b013e31820f77a0

[B2] D'Astous JL Sanders JO Casting and traction treatment methods for scoliosis Orthop Clin North Am 2007 38 4 477 484 v 10.1016/j.ocl.2007.03.006 17945127

[B3] Yazici M Non-Idiopathic Spine Deformities in Young Children

